# Public attitudes towards genetically modified polled cattle

**DOI:** 10.1371/journal.pone.0216542

**Published:** 2019-05-10

**Authors:** Emilie McConnachie, Maria Jose Hötzel, Jesse A. Robbins, Adam Shriver, Daniel M. Weary, Marina A. G. von Keyserlingk

**Affiliations:** 1 Animal Welfare Program, Faculty of Land and Food Systems, University of British Columbia, Vancouver, BC, Canada; 2 Laboratorio de Etologia Aplicada e Bem-Estar Animal, Departamento de Zootecnia e Desenvolvimento Rural, Universidade Federal de Santa Catarina, Florianópolis, Brazil; 3 Wellcome Centre for Ethics and Humanities, Nuffield Department of Population Health, University of Oxford, Oxford, United Kingdom; Universidade do Porto Instituto de Biologia Molecular e Celular, PORTUGAL

## Abstract

Genetic modification of farm animals has not been well accepted by the public. Some modifications have the potential to improve animal welfare. One such example is the use of gene editing (i.e. CRISPR (clustered regularly interspaced short palindromic repeats)) to spread the naturally occurring *POLLED* gene, as these genetically hornless animals would not need to experience the painful procedures used to remove the horns or horn buds. The aim of the current study was to assess public attitudes regarding the use of GM to produce polled cattle. United States (US) citizens (n = 598), recruited via Amazon Mechanical Turk, were asked “Do you think genetically modifying cows to be hornless would be…”, and responded using a 7-point Likert scale (1 = a very bad thing, 4 = neither good nor bad, 7 = a very good thing). Participants were then asked to indicate if they would be willing to consume products from these modified animals. We excluded 164 of the original 598 participants for not completing the survey, failing any of three attention check questions, or providing no or unintelligible qualitative responses. Respondents were then asked to provide a written statement explaining their answers; these reasons were subjected to qualitative analysis. Comparison of Likert scale ratings between two groups was done using the Wilcoxon rank-sum test, and comparisons between more than two groups were done using the Kruskal-Wallis rank test. More people responded that the modification would be good (Likert ≥ 5; 65.7%) than bad (Likert ≤ 3; 23.1%), and that they would be willing to consume products from these animals (Likert ≥ 5; 66.0%) versus not consume these products (Likert ≤ 3; 22.6%). Qualitative analysis of the text responses showed that participant reasoning was based on several themes including animal welfare, uncertainty about the technology, and worker well-being. In conclusion, many participants reported positive attitudes towards GM polled cattle; we suggest that people may be more likely to support GM technologies when these are perceived to benefit the animal.

## 1. Introduction

The field of genetic engineering has been revolutionized by the development of universal designer nuclease systems that allow for precise knock-ins and knock-outs of genetic material (e.g. clustered regularly interspaced short palindromic repeats [CRISPR], transcription activator-like effector nucleases [TALENs], and zinc finger nucleases [ZFNs]; [1]). These technologies provide users greater control over gene modifications and broaden the potential applications of genetic modification [1]. While there is currently only one gene modified (GM) animal approved for consumption (AquAdvantage Salmon; [2]), research involving genetically modified agricultural animals has been ongoing for over a decade. Genetic modification of farm animals has been targeted to reduce the environmental impact of agriculture (e.g. decreased phosphorous production from pigs; [3]), improve production (e.g. increased muscle mass in cattle; [4]), enhance meat quality (e.g. improved nutrient composition in pork; [5]), and improve animal welfare (e.g. resistance to diseases such as avian flu in chickens; [6]).

One application of gene modification with the potential to benefit animals is the creation of hornless dairy cattle. Dairy cattle typically grow horns, and these horns are often removed at a young age using a procedure called disbudding (or dehorning if the procedure is done on older animals) with the aim of reducing the risk of horn-related injury to workers and other animals. Horn buds can be removed using several methods including surgical scooping and cautery [7]. Regardless of the method, there is evidence that calves experience considerable pain during and after the procedure. This pain can be mitigated with a local anesthetic and non-steroidal anti-inflammatory drugs [7], but only 18 to 28% of operations in the United States use pain relief when dehorning calves [8–10].

Not all cattle grow horns; a single, naturally occurring and dominant gene (*POLLED*) is responsible for this hornless trait. This gene can be spread using traditionally breeding techniques, but traditional breeding is a slow process. Carlson et al. [11] used TALENs to insert the *POLLED* gene into the genome of Holstein fibroblasts, resulting in the creation of two healthy, hornless calves. This genetic modification could provide a way to rapidly spread this hornless trait, eliminating the need for dehorning.

Previous work has shown public opposition to genetically modifying animals used for food (see [12] for a review) although Americans tend to be more accepting than Europeans [13]. Genetically modifying animals is considered less acceptable than genetically modifying plants [14,15] but both are widely opposed (e.g. [16]). In one survey, the public rated genetically engineering animals as the most negative of various food-technologies, including the use of pesticides and hormones [15]. At the same time, other work has shown disapproval of dehorning in the absence of pain mitigation [17]. Thus, a genetic modification that improves welfare by eliminating the need for a painful surgery may be perceived positively. More generally, little work has examined how people evaluate gene modification methods when these have the potential of improving animal welfare. The aims of this study were to explore public attitudes, and the underlying reasons for these attitudes, towards the use of gene modification to produce polled cattle to improve animal welfare.

## 2. Materials and methods

The University of British Columbia Behavioural Research Ethics Board (H17-01354) approved this study.

### 2.1. Survey sample and design

Five hundred and ninety-eight US citizens were recruited to take a survey using Amazon Mechanical Turk (www.mturk.com). We excluded 42 participants for not finishing the survey, 98 participants for failing any of three attention check questions, and 24 participants for providing no or only unintelligible qualitative input. The final sample consisted of 434 participants.

Amazon Mechanical Turk provides a sample of individuals that are more diverse than groups generated from standard Internet samples while maintaining comparable reliability [18–20]. To avoid self-selection bias, participants were not informed of the specific nature of the survey prior to participating. Participants were simply asked if they would be willing to “take a survey about technology and agriculture.” The survey instrument assessed familiarity, general attitudes, perceived risk, perceived benefit, willingness to consume, and knowledge of the techniques. These factors are commonly evaluated when investigating attitudes towards technology used in agriculture (e.g. [21]).

After consenting, participants were instructed that they would be participating in a study about genetic engineering and that, “*Genetic modification is the process of using biotechnology to alter the genetic information (DNA) of an organism to produce a certain trait*.*”* They were then asked three questions designed to assess their familiarity with the topic (“*How much have you heard or read about*…” “*horn removal in cattle*”, “*genetic modification*”, and “*genetically modifying cows to be hornless*”). Responses to all three items were collected using a 7-point Likert scale (1 = *nothing at all*, 7 = *a great deal*). Participants then read the following prompt:

“*Almost all dairy cattle in the United States have their horns removed because horns pose a potential danger to workers and other cattle*. *Typically*, *horn removal occurs when the animals are young and involves burning or cutting out horn-growing tissue*. *This process is painful and about 80% of the time no pain-killing drugs are used to minimize this pain*. *An alternative to horn removal is genetically modifying cattle so that they never grow horns to begin with*. *This method involves integrating a ‘hornless’ gene (which is found in some breeds of cattle) into the cow genome and results in all calves being born without horns*.”

After reading the information, participants were asked, “*Do you think genetically modifying cows to be hornless would be*…” and responded using a 7-point Likert scale (1 = *a very bad thing*, 4 = *neither good nor bad*, 7 = *a very good thing*). They were also asked to explain the reason(s) for their rating in a text box. There was no text limit to this open-ended response.

Perceptions of risks and benefits were assessed using two items. Participants were first asked to rate how risky and beneficial they perceived genetically modifying cattle to be hornless (1 = *not at all risky*, 7 = *very risky*; 1 = *not at all beneficial*, 7 = *very beneficial*); no distinction was made in the prompt regarding what aspects of gene modification they viewed as being risky or beneficial. Following this we included two measures of behavioural intention. Participants were asked if they would personally be willing to consume food products from GM hornless cattle (1 = *strongly disagree*, 7 = *strongly agree*), followed by an item asking them if they believed most Americans would be willing to consume these products (1 = *strongly disagree*, 7 = *strongly agree*). This type of indirect measure has been used to assess the presence of social desirability bias [22,23]. Knowledge about the process of gene modification was assessed using five items modified from Hallman et al. [24]. The number of correct responses (ranging from 0 to 6) was used to create a knowledge score. Finally, participants answered a series of standard demographic questions including age, gender, education, ethnicity, political and religious affiliations, living environment (i.e. urban versus rural), and income, as well as some project-specific demographic questions such as dietary preferences, perceived importance of animal welfare, and number of times they had visited a dairy or beef farm in the past year. Upon completion, participants were debriefed, thanked, and paid $0.60.

### 2.2 Quantitative analysis

Stata IC15.0 (StataCorp LP, College Station, TX) was used for statistical analysis, and a P-value < 0.05 was considered significant. To assess correlations between two ordinal ratings as well as between measures on an ordinal and a continuous scale, Spearman’s rank correlation coefficients were calculated. Comparison of Likert scale ratings between two groups was done the Wilcoxon rank-sum test (Z value reported), and comparison between more than two groups was done using the Kruskal-Wallis rank test (χ2 value reported).

### 2.3 Qualitative analysis

Representational thematic text analysis was applied to the open-ended responses [25]. Three individuals trained in qualitative research read 50 randomly selected responses and independently identified the themes present in these responses. Three trained individuals (including E.M., M.J.H, and a third individual, C. Cardoso) were used to minimize researcher bias and increase the likelihood that all themes present in the responses were recognized. These researchers then came together and discussed their findings, identified and debated discrepancies, and ultimately compiled a document detailing the final, agreed-upon themes. Themes were developed inductively. After developing the themes, responses were coded in batches. First, two of the three authors (E.M. and M.J.H) independently coded approximately 20% (n = 73) of the total available responses according to these themes. Discrepancies between coding decisions were discussed and coding was finalized and repeated for this batch of responses. This process was repeated for half of the remaining responses (i.e. 40% of the original responses; n = 180), with any discrepancies resolved through discussion. Finally, E.M. coded the remaining 181 responses (i.e. 40% of the original responses).

## 3. Results

### 3.1 Quantitative findings

The mean (± SD) age of respondents was 36 (± 12) years old, 56% of respondents were male, and 80% were white. Other demographics are reported in Table 1. Compared to US census data, the study sample was of similar age but more educated, less religious, more liberal, and more likely to be male and white [26–30]. Mean survey completion time was 4.9 (± 2.3) min. A majority of respondents (80%) reported having heard or read “nothing” about genetically modifying cows to be hornless and 61% had heard or read “nothing” about the practice of dehorning cows.

**Table 1:** Demographics of the survey participants (n = 434). All individuals were American, over 18 years old, and users of Amazon Mechanical Turk.

Demographic Variable	Value
**Education level**	
Less than 4 yrs of post-secondary education	51%
At least 4 yrs of post-secondary education	49%
**Household pre-tax income**	
Less than $35,000	34.6%
$35,000-$74,999	38.0%
At least $75,000	27.4%
**Political affiliation**	
Liberal	54.8%
Centrist	17.5%
Conservative	27.7%
**Religious**	35.3%
**Living environment**	
Urban / suburban	83.0%
Rural	17.1%
**Region**	
West	25.3%
South	38.6%
Midwest	19.2%
Northeast	16.8%
**Pescatarian, vegetarian, or vegan**	6.5%
**Perception of importance of animal welfare**	
Not important / slightly important	8.1%
Moderately important	25.8%
Very important / extremely important	66.1%
**Visited dairy or beef farm in past year**	16.6%

**Fig 1:**
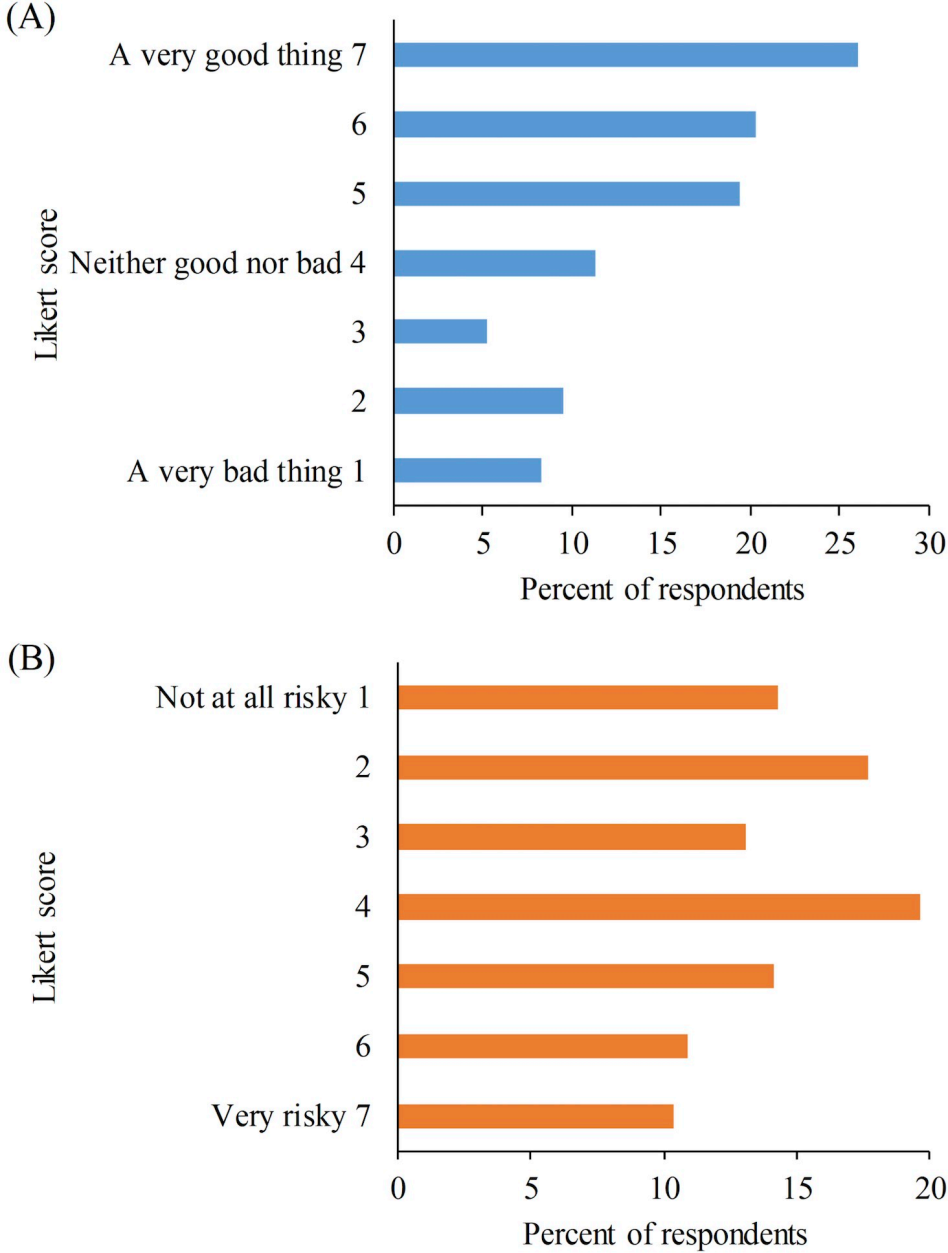
Participant (n = 434) responses regarding (A) attitudes and (B) perceived risk of genetically modified polled cows. Attitudes were determined from the question “Do you think genetically modifying cows to be hornless would be…”. Perceived risk was assessed by asking “How risky do you think genetically modifying cows to be hornless is?”.

**Fig 2:**
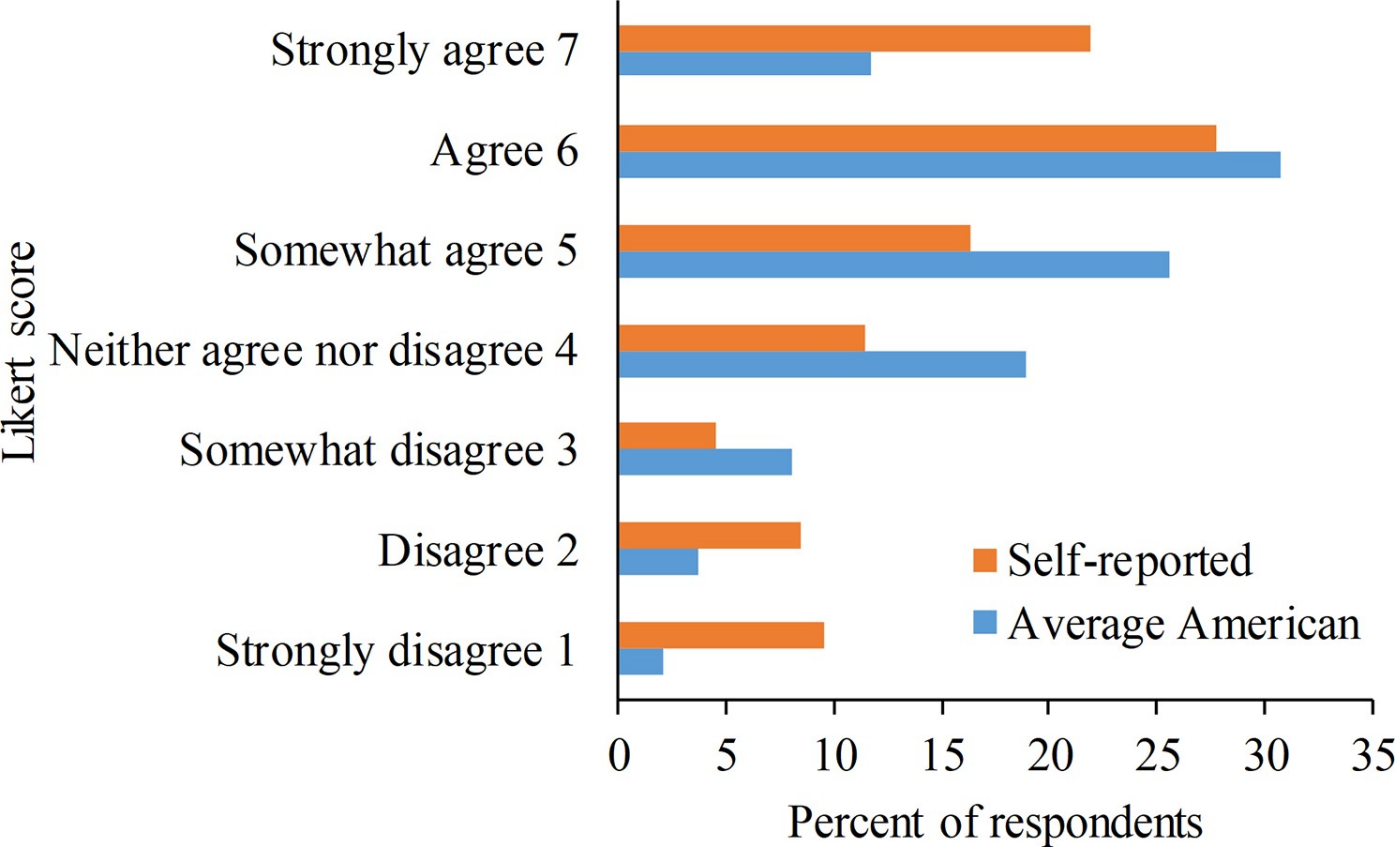
Participant (n = 434) responses regarding willingness to consume products from genetically modified polled cows. Willingness to consume was determined by asking participants “Please tell us how much you agree or disagree with the following statements” followed by questions stating “*I am willing* to consume food products from cows genetically modified to be hornless” (Self-reported) and “*Most Americans* are willing to consume food products from cows genetically modified to be hornless” (Average American).

In total, 66% of participants responded that genetically modifying cattle to be hornless was a good thing (Likert ≥ 5; Fig 1) and 66% of participants indicated they were willing to consume products from cattle genetically modified to be hornless (Likert ≥ 5; Fig 2). Twenty-three percent of participants responded that this gene modification was a bad thing (Likert ≤ 3; Fig 1) and 23% said they were not willing to consume these products (Likert ≤ 3; Fig 2).

Mean (± SD) attitude was 4.9 (± 1.9). Self-reported willingness to consume was also 4.9 (± 1.9), and these two measures were positively correlated (r_s_ = 0.77, p < 0.001). Self-reported willingness to consume and the participant’s judgment of “Most Americans” willingness to consume (Mean: 5.0 ± 1.4) were also positively correlated (r_s_ = 0.40, p < 0.001).

Overall, participants perceived lower risks (Mean = 3.8 ± 1.9) than benefits (Mean = 4.8 ± 1.8) of genetically modifying cattle to be hornless. Risk and benefit perceptions were negatively correlated (r_s_ = -0.64, p < 0.001). Perceived benefits were associated with positive attitudes (r_s_ = 0.83, p < 0.001) and greater willingness to consume (r_s_ = 0.68, p < 0.001); whereas, perceived risks were associated with negative attitudes (r_s_ = -0.72, p < 0.001) and lesser willingness to consume (r_s_ = -0.73, p < 0.001).

On average, participants answered four of the five genetics knowledge questions correctly. Higher scores on the knowledge questions were associated with more positive attitudes (r_s_ = 0.16, p < 0.001) and greater willingness to consume (r_s_ = 0.17, p < 0.001). Attitudes varied in relation to gender, with males tending to hold more positive attitudes (z = 2.6, p = 0.01) and greater willingness to consume (z = 4.3; p < 0.001). Religious participants tended to hold more negative attitudes (z = 1.8, p = 0.08) and expressed lower willingness to consume (z = 4.0, p < 0.001). Participant age was negatively associated with their attitudes (r_s_ = -0.11; p = 0.02) and willingness to consume (r_s_ = -0.13; p = 0.005). Political affiliation was not associated with attitude, but was with to willingness to consume (χ^2^ with ties = 6.1, 2 df, p = 0.049), with liberals having the highest attitude scores. Neither attitude nor willingness to consume were associated with income, education, region, whether the participant had visited a farm, or living environment (all p’s > 0.05); these factors are not discussed further.

### 3.2 Qualitative results

**Fig 3:**
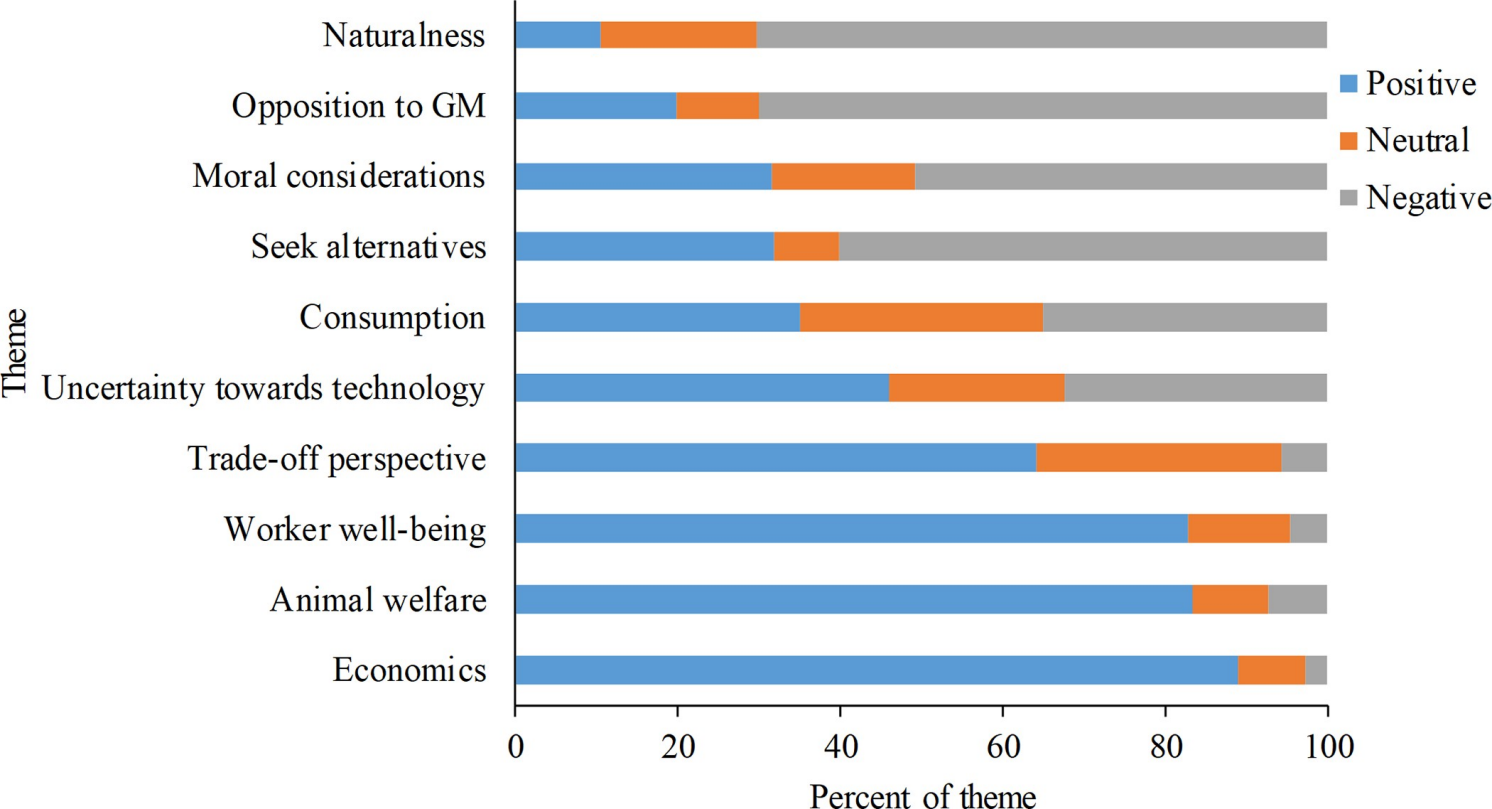
Attitude prevalence within themes. Attitudes were calculated from responses to a question “Do you think genetically modifying cows to be hornless would be…” and responded using a 7-point Likert scale (1 = a very bad thing, 7 = a very good thing). For graphical purposes responses were categorized as positive [5–7], neutral [4], and negative [1–3].

Ten themes emerged from the open-ended questions. In order of prevalence, these themes were 1) animal welfare, 2) uncertainty towards technology, 3) worker well-being, 4) moral considerations, 5) trade-off perspective, 6) seeks alternative, 7) naturalness, 8) opposition to GM, 9) economics, and 10) consumption (Table 2). All themes were raised among all response types (support, neutral, oppose) though some themes were more prominent among certain response types than others (Fig 3).

**Table 2:** Open-ended responses to the question “Please explain your response”.

Theme	Issues discussed in responses	Responses (%)*
Animal welfare	Cow or future generations’ well-being, pain, quality of life, treatment, health, affect, etc.	72.4
Uncertainty towards technology	Further testing, licensing, or experimentation, weariness of unknown side-effects, or other outcomes of using GM technology	17.1
Worker well-being	Farm-worker safety, job satisfaction, well-being, and happiness	14.8
Moral considerations	Moral considerations including those based in religion, morality, duty, personal beliefs, and ethics	14.5
Trade-off perspective	Responses that discussed both positive and negative aspects of GM polled cows, weighing them against each other	12.0
Seeks alternative	A preference towards pursuing an avenue other than current dehorning practices or gene modification	11.5
Naturalness	Natural processes, nature, or the naturalness/unnaturalness of the proposed situation	10.8
Opposition to gene modification	A dislike, disapproval, distrust, or other negative view towards gene modification as a whole or applied to the food system	9.2
Economics	Cost, value, profit, etc. for farmers, scientists, consumers, etc.	8.3
Consumption	Quality of food products, including taste, nutrition, texture, and safety and the health, well-being, and safety of consumers	8.1

* Responses could contain more than one theme, so the percentages shown below do not sum to 100.

#### 3.2.1 Animal welfare

Responses in the theme “animal welfare” discussed factors such as pain, quality of life, humane treatment, health, and affect. A consideration of many participants was the painfulness of dehorning and the humaneness of the procedure (e.g. “Certainly [cattle] must feel the pain when their horns are removed. It would therefore be a civilised and humane action to genetically modify the cows to be born hornless, to spare this them unnecessary pain” [R320]). Others considered the welfare impacts of GM polled cattle on other animal welfare and safety issues, for example, “the cattle are being bred for a purpose… if [GM] keeps workers safe as well as other cattle–go for it” [R160]. Some respondents did not discuss specific aspects of welfare, but rather considered the animal’s general quality of life (e.g. “we might as well make their lives a little better” [R133]). The impact of dehorning on cattle health was discussed by some participants (e.g. “removing [horns] opens up routes to infection” [R215]) and the impact of gene modification on cattle health was discussed by others (e.g. “I feel this is a modification that would be good for the health of the cattle” [R212] and “with genetically modifying animals, we don’t truly know what kind of side effects may happen to the long term health of [the] animal” [R305]). There were also participants who considered the inherent value of the horns to cattle (e.g. “I would worry about [cattle] losing any potential benefits to having horns” [R273]).

#### 3.2.2 Uncertainty towards technology

The theme “uncertainty towards technology” included responses that directly expressed some aspects of concern towards gene modification or biotechnology including a desire for more testing, a fear of consequences, or other measures of wariness. The potential for unintended side effects was often discussed (e.g. “you never know, but there is always a possibility of unintended consequences somewhere down the road” [R418]). A desire for more testing was a commonly expressed (e.g. “if it can be proven that there are no harmful side effects to the breed then I think it is OK” [R121] and “I think there needs to be rounds and rounds of testing before it becomes the norm” [R62]). The general efficacy of gene editing technologies was discussed (e.g. “Sometimes you can really botch a genome when you splice in DNA, and changing something as drastic as whether or not [cows] have horns sounds like quite the undertaking.” [R165]).

#### 3.2.3 Trade-off perspective

This theme encompassed responses that considered both positive and negative aspects of genetically modifying polled cows. These responses “saw both sides” of the proposal by considering arguments for it and against it. For example, R372 says, “It would be a convenience for dairy workers, but I don't think it's safe to alter the genes of animals without understanding how it might affect them in the future”. Here, R372 discusses both the benefit to the workers (a positive consideration) and their uncertainty about the technology and long-term animal well-being (negative considerations). Worker well-being, doubts about technology, and animal welfare are thus all themes present in this response. These trade-off analyses provide unique insight into the thought process of respondents as well as the propensity to critically think and evaluate multiple sides of an argument. Although respondents touched on other themes through their trade-off evaluation, as exemplified above, almost every response in this theme discussed animal welfare, uncertainty towards technology, opposition to GM, and moral considerations were present in approximately a quarter of responses. Overall, participants who fell into this theme displayed critical thinking and expressed a diverse set of thoughts.

#### 3.2.4 Worker’s well-being

Many participants considered the safety of workers to be important (e.g. “It would still accomplish what is needed to be done for safety reasons, but without harm/pain to the animal” [R337]). Some respondents thought management should be altered, so that cattle do not need to be dehorned or genetically modified to improve worker safety (e.g. “it doesn’t change the root cause of the problem, which is that factory farming is dangerous to the animals and workers” [R351]). Many respondents discussed the reduction in work that GM polled cattle would provide farmers (e.g. “it would prevent extra work involved with having to remove the horns in young cattle” [R178] and “it would save dairy cattle workers time and energy” [R200]).

#### 3.2.5 Moral considerations

The theme encompassing ethical qualms, religious considerations, ideas of right and wrong, and other moral ideas was collectively called “moral considerations” (Table 2). Responses in this theme touched on a vast array of ideas, from plant vs. animal considerations (“I can understand when it comes to plants, but animals are conscious, and it doesn't feel morally right for me to support that.” [R353]), to discussion of overextended human power (“I think it is ‘playing God’ and is immoral and unethical” [R184]), to consideration of duty to animals (“if we were to make [meat production] even slightly less painful for the animals, [animal agriculture] would be less ethically reprehensible” [R345]). Many participants noted that this “seemed wrong” or “felt wrong” with further explanation.

#### 3.2.6 Seek alternative

The “seek alternative” theme included responses where a desire for an alternative option to the current dehorning practices or using genetic modification to produce polled cattle was discussed. This theme had three popular responses: 1) a direct questioning of current dehorning practices (“I do question the practice of cutting the horns off at all. Could they not find a different solution?” [R44]), 2) a desire to not remove the horns (“In an ideal world, we would leave the cows and their horns alone, period.” [R422]), and 3) a preference for legally enforcing the use of pain mitigating drugs during dehorning surgery (“I think instead it would be more beneficial to legally require the use of pain killing drugs for horn removal.” [R30]). Some participants discussed a preference for selectively breeding polled cattle (“I can understand breeding the [horned] cows to hornless cows so that farther down the line, they all become hornless” [R189]). Other responses discussed a preference for eliminating factory farming (“it’s definitely treating the symptom rather than the disease of animal factory farming, so it seems like a waste of resources and a distraction away from the real problems of animal agriculture” [R153]), and changing management styles (“animals should not be altered to make it easier for humans to manage. Humans should determine safer ways to handle these animals, should they continue to be farmed for consumption” [R316]).

#### 3.2.7 Naturalness

A number of respondents discussed nature, natural processes like evolution, and naturalness in their responses. It was common for participants in this category to appeal to nature as being the reason for their earlier responses (“it is unnatural” [R186] and “We should not be interfering with nature in such a way. There will most likely be grave consequences.” [R128]). Others noted “unnaturalness” but did not object to this (e.g. “It seems so unnatural to me to do this to the cows but I do not believe something being unnatural is a ‘bad’ thing.” [R170]). Some participants discussed the existence of the hornless gene in non-GM cows (e.g. “I am generally dubious of genetic modification, but in this case, we’re simply using genes cows already have and putting them in ALL cows” [R207]). Domestication and selective breeding were talked about as well (e.g. “I don’t see any particular reason not to do this… cows have been domesticated to such a large extent, over such a large period of time, that there’s really nothing left natural about them to begin with… I don’t see any problem with it” [R46]). Lastly, some people discussed evolution (e.g. “I think evolution should tell us what a creature should be and look like, and we shouldn’t play with that just to suit our industries or plans” [R39]).

#### 3.2.8 Opposition to GM

The themes “opposition to GM”, “uncertainty towards technology”, and “seek alternatives” all suggested some discontent with gene modification. “Opposition to gene modification” was the most direct labelling of this dissatisfaction. Some respondents in this category solely considered opposition towards gene modification to explain their attitude towards GM polled cattle, such as Respondent 125 who said “modifying the DNA of anything is a very bad idea”. Others held their opposition to be one of multiple considerations; for example, “I'm not onboard with genetic modification normally, but to do so in order for the animal not to be in pain” [R138].

#### 3.2.9 Economics

Some participants mentioned efficiency or finances in regards to the genetically modified polled cattle. Genetically modifying cows to be polled was considered more efficient than dehorning them by some participants (e.g. “rather than putting the cows through the pain and having someone have to cut a horn and possibly feel bad, it would just be more efficient to change the genes” [R150]). Some participants felt that producers and consumers would benefit financially from these animals through reduced labor and dehorning costs (e.g. “Thinking about it economically, the dairy cattle that are genetically modified to be hornless would automatically not require labor and capital to dehorn the cattle” [R26] and “not having [dehorning] would also reduce the cost (in man-hours and tools) of each head, and hopefully reduce the consumer’s cost” [R57]). However, other participants thought GM polled cows might be economically challenging (“Genetically modifying the animals sounds like it will be expensive” [R44]).

#### 3.2.10 Consumption

Many responses expressed concerns relating to the safety of milk and beef products derived from the GM polled dairy cattle (e.g. “Genetically modifying cows… may cause unknown side-effects which could potentially be harmful for the animal and the consumer of the animal” [R137] and “will the cow's milk or meat be fit for human consumption?” [R390]). Food quality was discussed, especially in regards to the taste of milk and meat products, as was the nutritional value of the food products (e.g. “could [genetic modification] change the taste or nutritional content of the milk produced?” [R160]).

## 4. Discussion

Previous research has investigated public perceptions regarding GM animals, but not in regards to specific applications of gene modification that have the potential to improve animal welfare. The current study provides the first exploration of perceptions towards GM polled cattle. When positioned from the perspective that this technology could eliminate the need for a painful procedure, participants in this study expressed predominantly positive attitudes towards. This finding contrasts previous work showing predominantly negative attitudes towards gene modification of animals [13,15,16,31–33]. As previous work has noted [34], risk and benefit perceptions were inversely related, and many of our participants perceived moderate to high benefits, arguably because they believed that animal welfare would be improved in the GM polled cattle.

The open-ended responses of our participants provide some insight into their reasoning, but no one theme was exclusive to any one attitude (or vice-versa). Animal welfare was the most commonly expressed theme and included concern for farm animal emotional and physical well-being as well as the desire to promote better animal welfare. This result aligns with other research suggesting that the public values farm animal welfare [35,36]. Many participants described current dehorning practices as inhumane, cruel, or painful for the animals; disapproval of dehorning without pain mitigation was also reported by Robbins et al. [17].

Commonly perceived risks of genetically modified food products include unknown long-term or unintended effects on consumer health, animal welfare, and the environment [13]. The open-ended responses indicated that our participants perceived these risks, although concerns for environmental impact were less often expressed. As noted in previous studies on attitudes towards GM foods, participants often expressed concerns about the impact of GM on animal welfare and humans consuming products from these animals [12,13,36]. There was clear desire for long-term testing on GM polled cattle to provide better data on the potential effects the GM could have on both humans and animals.

Some participants discussed the concept of naturalness, moral dilemmas, and ethical reasoning in their responses. The “unnaturalness” of gene modification has been previously noted as a basis for, and predictor of, objection to gene modification [37–39]. However, this statement remains contentious given that there is no clear consensus on what the argument of unnaturalness entails (see [40] for an overview). Some participants noted moral objections similar to those found in other studies, such as the idea that genetically modifying animals reinforces the idea that livestock are instruments for human use [41] and that humans should not “play God” [42]. Respondents also suggested that farmers have a duty of care to minimize animal pain and suffering, through either gene modification or alternative management. A similar finding was noted by Cardoso et al. [43]; participants in that study viewed farmers as failing in their duty of care towards the cow if they did not provide cows protection from heat stress.

Many of our respondents seemed to recognize both benefits and risks to the technology, with some detailed responses illustrating how people struggle with this trade-off. Cardoso et al. [43] also found evidence of participants considering such trade-offs when they considered different dairy cattle housing scenarios that emphasised different aspects of animal welfare (i.e. pasture as being more natural, and indoor housing being able to protect animals from heat stress); participants were more supportive of the scenario that was less ‘natural’ if this prevented heat stress.

Presenting a specific example of a GM animal to participants may provide more practical insight into public perceptions than asking participants about GM animals in general. Much of the existing literature evaluates perceptions towards “GM animals” in an abstract manner—investigating perceptions about the *idea* of GM animals rather than perceptions about specific applications. Here, an explicit example of GM animals and information on why this gene modification is relevant was provided to participants. Our approach likely allowed participants to more specifically evaluate the GM animal and, consequently, express attitudes that better represent how the gene modification of interest would actually be perceived if it were to progress into the food chain. However, as discussed above, many participants showed nuanced thinking, identifying numerous themes and considerations in their responses.

Studies exploring public attitudes may be subject to social desirability and self-selection biases. Social desirability bias can result in data that is skewed to show greater acceptance among participants than there actually is. To try to control for this, participants were asked what they thought of “Most Americans’” willingness to consume, as this approach shifts the social consequences of the participant’s response to someone else [22]. This question served as a measure that could be compared against the participant-focused willingness to consume question. The similarity in self-reported willingness to consume and the participants’ judged of the willingness of “Most Americans” suggests that social desirability bias was minimal.

This study has a number of limitations. First, participants were recruited via Mechanical Turk. Although this provides a useful convenience sample, the results should not be considered representative on a regional or national scale. Amazon Mechanical Turk provides a practical, cost-effective, and efficient way to gather a diverse group, but the survey sample is not representative of the American public, so care is required in making inferences regarding a wider population. Second, when the survey was distributed, the USDA’s most recent dairy report on cattle management [10] had not been released and thus other sources were used [8,9,44] to deduce the prevalence of producers using pain mitigation during dehorning. Thus, participants were informed that 20% of producers use of pain mitigating strategies during dehorning, which is lower than the most recent USDA report of 28% of producers. Reporting this somewhat higher number may have softened opposition to existing practice, and thus made participants less accepting of the GM alternative. Lastly, it is important to note that authors all work on the topic of animal welfare; this is likely to have influenced the tone of this manuscript and the direction of our research.

## 5. Conclusion

The majority of participants reported positive attitudes towards GM polled cattle and a willingness to consume products from these animals. Participants often discussed issues related to animal welfare in support of their views. These results suggest that participants may be more likely to support GM technologies when these are perceived to benefit animal welfare.

## Supporting information

S1 File McConnachie et al data. (CSV)

S2 File Copy of survey prepared using Qualtrics and launched using the Mechanical Turk platform: EM survey flow_0718.pdf, such as S1 File and S2 File. (PDF)
