# Supplementary material for: Public attitudes towards genetically modified polled cattle
Source: PLoS One. 2019 May 10;14(5):e0216542. doi: 10.1371/journal.pone.0216542 (PMC6510451; doi:10.1371/journal.pone.0216542)
Supplement: S2 File — (PDF) [file pone.0216542.s002.pdf]

GM Polled Survey - Emilie

1- Opening intro pre-start block Block Options

Q1

Consent

Principal Investigator: Dr. Marina von Keyserlingk, Professor and NSERC Industrial Research Chair, University of British Columbia, marina.vonkeyserlingk@ubc.ca 604.822.4898

Co-Investigator: Jesse Robbins, Post-Doctoral Fellow, University of British Columbia, 604.822.5715

Funding
This research is funded by the University of British Columbia.

Recruitment
Participation is this project is open to anyone over 18 years of age. You were recruited to participate in this study using Amazon's Mechanical Turk service.

Risks
There are no known risks in taking this survey. You may leave at any point during the survey.

Confidentiality
We will not collect any information that allows us to identify or contact you individually. The data you provide will be stored in a secure database and will only be accessible to the research team. This online survey is hosted by Qualtrics, a web survey company located in the USA. If you choose to participate, in the survey you understand that your responses will be stored in the USA. The privacy and security policy of the survey service provider can be found at: http://www.qualtrics.com/privacy-statement/.

If you have any concerns or complaints about your rights as a research participant and/or your experiences while participating in this study, contact the Research Participant Complaint Line in the UBC Office of Research Ethics at 604-822-8598 or if long distance e-mail RSIL@ors.ubc.ca or call toll free 1-877-822-8598.

Consent
I am over 18 years of age and I understand my participation in this study is entirely voluntary and that I may choose to quit at anytime. I understand that any information I provide will be anonymous and will only be stored and analyzed for the purposes of this research.

YES, I consent

Add Block

Block 16 Block Options

Q45

Thank you for agreeing to participate in this survey!

Add Block

2- Thx and genetic modification Block Options

Q3

Genetic modification is the process of using biotechnology to alter the genetic information (DNA) of an organism to produce a certain trait. In the following survey, you will be asked for your opinions about a specific application of genetic modification.

Add Block

3- Familiarity Randomized Block Options

Q48

How much have you read or heard about ***horn removal in cattle*** (also known as dehorning or disbudding)?

Nothing at all	A little	A moderate amount	A lot	A great deal
○	○	○	○	○

Page Break

Q49

How much have you read or heard about ***genetic modification***?

Nothing at all	A little	A moderate amount	A lot	A great deal
○	○	○	○	○

Page Break

Q50

How much have you read or heard about ***genetically modifying cows to be hornless***?

Nothing at all	A little	A moderate amount	A lot	A great deal
○	○	○	○	○

Add Block

Block 14 Block Options

Q39

Please carefully read the following information so that you can answer several questions that follow.

Almost all dairy cattle in the United States have their horns removed because horns pose a potential danger to workers and other cattle. Typically, horn removal occurs when the animals are young and involves burning or cutting out horn-growing tissue. This process is painful and about 80% of the time no pain-killing drugs are used to minimize this pain. An alternative to horn removal is genetically modifying cattle so that they never grow horns to begin with. This method involves integrating a “hornless” gene (which is found in some breeds of cattle) into the cow genome and results in all calves being born without horns.

Q40

This question lets you record and manage how long a participant spends on this page. This question will not be displayed to the participant.

Add Block

5- CompCheck Randomized Block Options

Q71

To make sure you carefully read the previous information, please answer the following questions:

Q69

The majority of dairy cows in the United States have their horns removed at a young age.

- True
- False

Q70

It's impossible to genetically modify cows to be hornless.

- True
- False

Q35

Most of the time, cows are given pain killing drugs to lessen the pain associated with horn removal.

- True
- False

Add Block

4- Part 2: Introduction

Block Options

Q51

Almost all dairy cattle in the United States have their horns removed because horns pose a potential danger to workers and other cattle. Typically, horn removal occurs when the animals are young and involves burning or cutting out horn-growing tissue. This process is painful and about 80% of the time no pain-killing drugs are used to minimize this pain. An alternative to horn removal is genetically modifying cattle so that they never grow horns to begin with. This method involves integrating a "hornless" gene (which is found in some breeds of cattle) into the cow genome and results in all calves being born without horns.

Q46

In your opinion, do you think genetically modifying cows to be hornless would be:

A very bad thing			Neither good nor bad			A very good thing
○	○	○	○	○	○	○

Q53

Please explain your response:

Add Block

6- Risk-Benefit Randomized

Block Options

Q66

How risky do you think genetically modifying cows to be hornless is?

Not at all risky						Very risky
○	○	○	○	○	○	○

Page Break

Q68

How beneficial do you think genetically modifying cows to be hornless is?

Not at all beneficial						Very beneficial
○	○	○	○	○	○	○

Add Block

7- BehaveIntent   Block Options

Q41

Please tell us how much you agree or disagree with the following statements.

Page Break

Q64

I am willing to consume food products from cows genetically modified to be hornless.

Strongly disagree	Disagree	Somewhat disagree	Neither agree nor disagree	Somewhat agree	Agree	Strongly agree
○	○	○	○	○	○	○

Page Break

Q65

Most Americans are willing to consume food products from cows genetically modified to be hornless.

Strongly disagree	Disagree	Somewhat disagree	Neither agree nor disagree	Somewhat agree	Agree	Strongly agree
○	○	○	○	○	○	○

Add Block

8- Knowledge intro   Block Options

Q52

Some people tend to know very little about genetic modification, while others seem to know quite a lot. In the next section we want to see how much you know about genetic modification.

Add Block

8.1- Knowledge Q - fishy   Block Options

Q62

Cows modified with genes from a catfish would probably taste fishy.

- True
- False

Q57

How certain are you about your answer?

Not at all certain				Very certain
○	○	○	○	○

Add Block

8.2- Knowledge Q - modifypersonsgenes

Block Options

Q61

By eating a genetically modified product, a person's genes could also become modified.

- True
- False

Q55

How certain are you about your answer?

Not at all certain				Very certain
○	○	○	○	○

Add Block

8.3- Knowledge Q - containgenes

Block Options

Q80

Genetically modified cows contain genes while ordinary cows do not.

- True
- False

Q81

How certain are you about your answer?

Not at all certain				Very certain
○	○	○	○	○

Add Block

8.4- Knowledge Q - transfer genes possible

Block Options

Q84

It is possible to transfer plant genes into animals.

- True
- False

Q85

How certain are you about your answer?

Not at all certain ... Very certain

Add Block

8.5- Knowledge Q - bigger

Block Options

Q57

Genetically modified animals are always bigger than ordinary animals

- True
- False

Q58

How certain are you about your answer?

Not at all certain ... Very certain

Add Block

Demo

Block Options

Q44

Finally, we have a few questions about you.

Q5

Age

Q7

Gender

- Male
- Female

Q39

Which of the following best describes your ethnicity?

- White, Caucasian
- Black, African American
- Asian, Pacific Islander
- Mexican, Latino
- American Indian
- Other

Q42

What is the highest level of school you have completed or the highest degree you have received?

- Less than high school degree
- High school graduate (high school diploma or equivalent including GED)
- Some college but no degree
- Associate degree in college (2-year)
- Bachelor's degree in college (4-year)
- Master's degree
- Doctoral degree
- Professional degree (JD, MD)

Q38

Overall, how would you characterize your political views?

Very liberal	Liberal	Somewhat liberal	Centrist	Somewhat conservative	Conservative	Very conservative
○	○	○	○	○	○	○

Q37

Please indicate the answer that includes your entire household income in (previous year) before taxes.

- Less than $25,000
- $25,000-$34,999
- $35,000-$49,999
- $50,000-$74,999
- $75,000-$99,999
- $100,000-$149,999
- $150,000 or more

Q29

Do you consider yourself religious?

- Yes
- No

Q15

Which of the following best describes the area where you have lived most of your life?

- Urban
- Suburban
- Rural

Q13

Do you consider yourself a pescatarian, vegetarian, or vegan?

- Yes
- No

Show Discussion (0)

Q73

How important do you think animal welfare is?

Not important at all	Slightly important	Moderately important	Very important	Extremely important
○	○	○	○	○

Show Discussion (0)

Q74

In the past year, how many times have you been to a dairy or beef farm?

Never	1 time	2 times	3 times	More than 3 times
○	○	○	○	○

Add Block

End of Survey

Survey Termination Options...

Qualtrics.com Contact Information Legal
